# T Cell Roles and Activity in Chronic Sclerosing Sialadenitis as IgG4-Related Disease: Current Concepts in Immunopathogenesis

**DOI:** 10.1155/2022/5689883

**Published:** 2022-06-20

**Authors:** Hazim Mahmoud Ibrahem

**Affiliations:** Department of Basic Sciences, Al-Mustansiriyah University, College of Dentistry, Baghdad, Iraq

## Abstract

IgG4-related disease is a multiorgan immunological fibroinflammatory disorder characterized by lymphoplasmacytic infiltration and fibrosis in multiple organs accompanied by high serum IgG4 levels. The salivary glands are the most common organs involved in this disease. Recently, chronic sclerosing sialadenitis affecting salivary glands, formerly known as Küttner's tumor, and Mikulicz's disease have been classified as a class of IgG4-related diseases. The etiopathobiology of IgG4-related disease is not fully understood. It has recently been hypothesized that the inflammatory and fibrotic process and the increased serum IgG4^+^ levels in IgG4-related disease are the result of an interaction between B cells and T helper cells, suggesting that T cells may play a key role in the pathogenesis of this disease. The aim of this review is to discuss the proposed roles of different T cell subsets in the pathogenesis of IgG4-related disease focusing on their roles in immunopathogenesis of IgG4-related sialadenitis.

## 1. Introduction

At the beginning of the 21st century, IgG4-related disease (IgG4-RD), also known as IgG4-related sclerosing disease, was recognized as a multiorgan disease by Japanese physicians [1]. IgG4-RD is characterized by lymphoplasmacytic infiltration and fibrosis in multiple organs accompanied by high serum IgG4 levels [2, 3]. The histopathological features of all affected organs are very specific with a similar appearance [4, 5].

The underlying pathogenesis of IgG4-RD remains largely unknown [6]. In addition to B cells, recent studies revealed the accumulation of several subsets of T lymphocytes and their cytokines in the peripheral blood and tissues of the affected organs in IgG4-RD patients. These T cells include T helper type 2 (Th2) cells, regulatory T (Treg) cells, follicular helper T (Tfh) cells, CD4^+^ cytotoxic T lymphocytes (CTLs), and CD8^+^ CTLs; these T cell subtypes could contribute to the immunopathogenesis of IgG4-RD through the activation of B cells and plasmacytes, enhancement of IgG4 class-switch recombination, development of ectopic germinal centers, and induction of fibrosis [7–10].

Many medical conditions that have long been viewed as confined to single organs are now considered a part of the IgG4-RD spectrum [11]. In the head and neck region, the most commonly affected organs are the salivary glands [12, 13]. Recently, identification of IgG4 positive (IgG4^+^) plasma cells in both Küttner's tumor, a type of chronic sialadenitis, and the so-called Mikulicz's disease, a form of Sjögren's syndrome, resulted in their classification among IgG4-RD group that affects salivary gland tissues, designated as IgG4-related sialadenitis (IgG4-RS) and IgG4-related dacryoadenitis and sialadenitis (IgG4-RDS), respectively [12, 14–17].

This review focuses on the proposed and theoretical roles of T cells in the immunopathogenesis of IgG4-RS.

## 2. IgG4-Related Diseases (IgG4-RD)

### 2.1. Definition of IgG4-RD

IgG4-RD is a newly recognized multiorgan systemic immune-mediated disorder characterized by a chronic fibroinflammatory condition [18, 19]. The underlying etiology and pathogenesis of IgG4-RD remain largely obscure. It can affect essentially any organ, causing mass-forming (tumefactive) lesions involving various tissues [18–20]. Before this condition was recognized as IgG4-RD, it was considered an organ-specific disease [21]. However, in 2003, because of the observation of elevated serum IgG4 levels and the infiltration of IgG4^+^ plasma cells and fibrosis in the affected organs, this disorder was classified clinically and histopathologically as a single group of diseases known as IgG4-RD [14]. Clinically, this disease more frequently affects males above 50 years of age [19, 22–24].

### 2.2. Affected Organs

IgG4-RD can involve any organ. The most commonly involved organs are the salivary glands, the lacrimal gland, and the pancreas; however, it can affect other organs such as the retroperitoneum, lymph nodes, aorta, arteries, breast, prostate, thyroid, pericardium, and skin [18, 25].

### 2.3. Histopathological and Serological Features of IgG4-RD

A characteristic feature of IgG4-RD is the presence of similar histopathology findings across all of the affected organs [4, 22–28].

The typical and specific histopathological features are as follows: a dense lymphoplasmacytic infiltrate enriched in IgG4^+^ plasma cells with IgG4^+^ plasma cell/IgG^+^ plasma cells at *a* > 40% ratio; an irregularly whorled fibrotic pattern referred to as “storiform fibrosis;” obliterative phlebitis involving partial or complete obliteration of medium-sized veins by lymphoplasmacytic cell infiltration, which appear as an inflammatory nodule next to a patent artery; mild-to-moderate tissue eosinophilia with the absence of granuloma or tissue necrosis; and hyperplastic ectopic germinal centers [4, 5].

IgG4-RD is often associated with elevated serum IgG4 levels, though this feature must be present for the final definite diagnosis. Yet, elevated levels of serum IgG4 have also been reported in other conditions [26, 27]. High number of IgG4^+^ plasma cells within affected organs cannot be utilized as the sole diagnostic parameter for IgG4-RD, as the diagnosis should be based on clinical, serological, and histological findings [27, 29].

### 2.4. Classification Criteria for IgG4-RD

In 2019, a new 3-step classification process system was proposed for the classification of IgG4-RD. First, a potential IgG4-RD case must involve at least one of the 11 possible organs consistent with IgG4-RD criteria. Second, the exclusion criteria, consisting of a total of 32 clinical, radiologic, serologic, and pathologic items, must be applied; the presence of any exclusion criterion excludes the patient from IgG4-RD classification. Third, eight inclusion criteria, including clinical findings, radiology assessments, serologic results, and pathology interpretations, are applied. American College of Rheumatology/European League Against Rheumatism (ACR/EULAR) classification criteria for IgG4-RD demonstrate excellent test performance and should contribute to future clinical, epidemiologic, and basic scientific investigations [30].

### 2.5. General Comprehensive Diagnostic Criteria of IgG4-RD

The revised comprehensive diagnostic (RCD) criteria for IgG4-RD in any involved organ were proposed by Umehara et al., which depend on three critical items: clinical, serological, and pathological findings in IgG4-RD patients, as listed in Table 1 [31].

Upon applying both the 2019 ACR/EULAR classification criteria and RCD criteria to a group of previously diagnosed IgG4-RD patients, Byung-Woo Y. (2020) proposed that there is agreement between these two sets of criteria for the diagnosis of IgG4-RD [32].

### 2.6. Etiology and Pathogenesis of IgG4-RD

The etiology and pathogenesis of IgG4-RD are not fully understood. Many factors seem to contribute to IgG4-RD development, including allergic, autoimmune, and genetic factors [33, 34]. An antigen-driven inflammatory condition or infection has been suggested based on the features of this disease, including the chronic clinical course, specific organ involvement, common histopathological features shared by different unrelated affected organs, and lastly their response to steroid treatment [35].

Furthermore, researchers in the field of immunology identified that certain abnormal immunological mechanisms, derived from both innate and adaptive immunity, are involved in the pathogenesis of IgG4-RD [20, 36].

### 2.7. T Cells in the Pathogenesis of IgG4-RD

In the last decade, several pathophysiological mechanisms responsible for the development of IgG4-RD have been described. Some of these suggested mechanisms indicate that B lymphocytes might act as functional antigen-presenting cells to CD4^+^ T cells, maintain CD4^+^ memory T cells by providing antigen-independent factors, and promote the proliferation of pathogenic T cells [33].

Recent immunohistochemical studies showed that CD4^+^ T cells are the most abundant and active cells within the lymphoplasmacytic infiltrate in the affected organ in IgG4-RD patients, suggesting that, in addition to B cells, T cells could be key players in IgG4-RD pathogenesis [6, 19, 27, 33, 37–39].

In patients treated with rituximab (RTX), B cell counts are effectively depleted; thus, apart from depleting B cells, RTX is likely to interfere with the more important B cell/T cell cross-talk processes through the elimination of a major B-cell type required for persistence of antigen presentation to T cells and for the maintenance of T cell activation, thereby indicating that B cell depletion during treatment ultimately revokes the production of proinflammatory and profibrotic cytokines such as IL-4, IL-13, and IL-10 by the pathogenically activated T cell populations [33]. Thus, T cells have been recognized as key players in disease pathogenesis, but their contributions to IgG4-RD remain to be clarified.

### 2.8. T Cell Subsets in the Pathogenesis of IgG4-RD

In IgG4-RD, CD4^+^ T helper cells are the most abundant cells within disease-involved tissues and show increased levels in peripheral circulation [7]. The CD4^+^ T helper cell subsets that play important roles in IgG4-RD pathogenesis include Th2 cells, Treg cells, Tfh cells, and CD4^+^ CTLs [20].

Kamekura et al. suggested that subsets of T helper cells that play roles in IgG4-RD pathogenesis could be organized into two main groups according to their activity, intensity, and abundance in the affected tissue [40]. The different T cell subsets involved in the pathogenesis of IgG4-RD are listed in Table 2.

### 2.9. T Cell Subsets, Features, and Proposed Roles in the Pathogenesis of IgG4-RD

#### 2.9.1. CD4^+^ Th1 in IgG4-RD

Th1 is the main IFN-*γ* producing cells, but in case of IgG4-RD conflicting results have been reported regarding the involvement of Th1 cells in producing IFN-*γ* in IgG4-RD pathophysiology [41]. Recent studies have revealed that cytotoxic CD4^+^ T cells or Tfh1 cells, rather than Th1 cells, are the most likely IFN-*γ*-producing cells in IgG4-RD [42, 43].

#### 2.9.2. CD4^+^GATA3^+^ Th2 in IgG4-RD

IgG4 is a Th2-dependent isotype that has a low affinity for target antigens and represents the lowest and rarest fraction of the IgG subclasses. IgG4 is accounting for only 3–6% of total IgG in normal serum [44].

Th2 cells, which produce interleukin-4 (IL-4), IL-5, IL-10, and IL-13, have the capacity to induce IgG4 class-switch recombination, thereby directing B cells to produce IgG4 [3, 45–47].

Most studies have indicated that IgG4-RD is characterized by the predominant activation of Th2 with the overexpression of their cytokines [3, 43–47].

Conflicting results have been reported regarding the involvement of Th2 cells in IgG4-RD immunopathogenesis. Most studies revealed that Th2 cells accumulate only in the blood of IgG4-RD patients, whereas other studies showed that the levels of cytokines produced by Th2 are frequently increased in the affected tissues and peripheral blood of IgG4-RD patients [41, 44, 46, 48–52].

Previous studies demonstrated that circulating Th2 memory cells accumulate in patients with IgG4-RD who have history of atopy but not among those without atopic disease [41, 48–54]. This finding may explain the eosinophilia and increased serum IgE levels, which occur in 40% of IgG4-RD patients [53]. A subset of nonatopic IgG4-RD patients has peripheral blood eosinophilia and elevated IgE levels, suggesting that these conditions may be related to the overexpression of Th2 cytokines rather than atopy [6, 11, 53].

CD4^+^ GATA3^+^ Th2 cells were found to be sparse in the salivary glands of patients with IgG4-RD, and the percentage of CD4^+^ GATA3^+^ Th2 cells in tissues in IgG4-RD does not seem to be correlated with other diagnostic parameters, such as serum IgG4 concentrations and the number of affected organs [41, 43, 55, 56].

All these findings indicate the involvement of Th2 cell and its cytokines in the pathogenesis of IgG4-RDS [43, 48].

#### 2.9.3. CD4^+^Forkhead BoxP3 (FoxP3)^+^ Regulatory T (Treg) Cells in IgG4-RD

CD4^+^FoxP3^+^ Treg cells play an important role in the regulation of self-tolerance and the maintenance of tissue homeostasis [57–59]. These cells are a source of IL-10 and transforming growth factor-*β* (TGF-*β*), which are the key cytokines involved in the differentiation of IgG4-producing B cells and in the fibrogenesis, respectively [3, 7, 44–47, 57, 58].

In IgG4-RD patients, the numbers of CD4^+^CD25^+^Foxp3^−^expressing Treg cells are significantly higher in the affected tissues and peripheral blood in comparison to the cell count in patients with other autoimmune and nonautoimmune diseases [6, 11, 45, 46, 57, 60, 61].

Regarding the levels of cytokines produced by Treg cells in IgG4-RD, an overexpression of the regulatory cytokines IL-10 and TGF-*β* has been reported [3, 45, 50, 62]. IL-10 is implicated in B cell-induced production of IgG4 antibodies (Abs), and TGF-*β* is a fibrogenic cytokine that may be involved in the promotion of fibrosis in IgG4-RD [11, 63, 64].

A recent study revealed only a marginal increase in circulating Treg cells that were identified as CD4^+^CD45RO^+^CD39^+^CD25^+^FoxP3^+^ Treg cells in IgG4-RD patients (T cells with CD45RO^+^ are considered memory types of CD4^+^ T cells) [55].

#### 2.9.4. CD4^+^CXR5^+^ Tfh in IgG4-RD

*(1) Immunobiological Function of Tfh Cells in IgG4-RD*. Tfh cells can participate in B cell differentiation through IL-21 and promote the formation of germinal centers and the production of IgG4 Abs [62, 65–68]. Additionally, they assist in the development of most high-affinity Abs and memory B cells, indicating that disease-specific Tfh cells promote specific class-switching recombination to IgG4 [65]. Activated circulating Tfh2 (cTfh2) cells, which have high expression of programmed cell death 1 (PD-1; i.e., PD-1^hi^ Tfh2 cells), help naïve B cells differentiate into plasmablasts and initiate production of IgG4 [42, 66, 69]. In disease conditions, the interaction between B cells and Tfh cells is essential for Ab production and plays an important role in Ab-driven diseases, such as IgG4-RD [62].

There are two sets of Tfh cells. The first set is the peripheral circulating cells which are of three subsets: cTfh1 cells that are known to secrete mostly IFN-*γ*; cTfh2 cells subset that secretes IL-4, IL-5, and IL-13; and cTfh17 cells subset that secretes primarily IL-17, IL-21, and IL-22, whereas the second set of Tfh cells is the tissue-infiltrating one [60, 65–68].

*(2) CD4^*+*^^*+*^Tfh in IgG4-RD*. Generally, in IgG4-RD, these cells are significantly increased in both peripheral blood and disease-affected organs [6, 9, 29, 60, 66, 67, 70].

*(3) Circulating Tfh (cTfh) Cells in IgG4-RD*. The number of cTfh cells is significantly higher in IgG4-RD patients than in healthy individuals [42] and cTfh cells in the peripheral blood reflect the degree of Tfh cell infiltration in the diseased tissue [70, 71]. Furthermore, the number of cTfh2 cells is positively correlated with serum IgG4 levels and the IgG4 : IgG ratio [66].

Akiyama et al. showed that the numbers of circulating Tfh2 cells and plasmablasts were increased in patients with active, untreated IgG4‐related disease [66]. Akiyama et al. (2016) reported that cTfh2 cells, but not cTfh1 or cTfh17 cells, induced the differentiation of naïve B cells into IgG4-producing plasma cells and promoted the production of IgG4 in IgG4-RD patients [42].

*(4) Tissue-Infiltrating Tfh in IgG4-RD*. The number of activated Tfh cells is increased in the salivary gland of IgG4-RD patients and is correlated with disease activity but not with serum IgG4 level [42, 67, 69, 70].

*(5) Tfh Cells and the Biological Treatment of IgG4-RD*. Grados et al. showed that CD4^+^CXCR5^+^PD1^+^ Tfh cells were normalized under RTX treatment [71]. However, Sohan et al. revealed that, after RTX treatment, Tfh cells and TGF-*β*-producing cells remained in the blood of IgG4-RD patients, suggesting that these cells might be risk factors for incomplete resolution of the inflammatory process [9].

Recent studies showed that, in contrast to serum concentrations of IgG4 Abs and IL-4, cTfh2 cell counts do not decrease after glucocorticoid treatment; additionally, the persistently circulating activated cTfh2 cells might contribute to disease relapse after ceasing treatment [66, 72].

#### 2.9.5. T Follicular Regulatory (Tfr) Cells in IgG4-RD

*(1) Tfr Characteristics and Functions in IgG4-RD*. Recently, a subpopulation of T regulatory (Treg) cells named Tfr cells that coexpress markers of both Treg and Tfh cells has been identified. Similar to Treg cells, Tfr cells are regulated by regulatory molecules such as Bcl6, PD-1, ICOS, Foxp3, CD25, CTLA-4, TGF-*β*, and IL-10 which promote B cell differentiation and GC development [73–76]. Similar to B cells and Tfh cells, Tfr cells express CXCR5 [73–75]. In collaboration and interaction with Tfh cells and/or B cells, these cells assist in the class-switch recombination of B cells and germinal center (GC) formation [40, 73, 74, 77].

*(2) Tfr in Autoimmune Diseases and IgG4-RD*. Studies have demonstrated that, in some immune-mediated diseases such as multiple sclerosis and systemic lupus erythematosus, there is a significant correlation between the percentage of Tfr cells and other clinical parameters [78].

In IgG4-RD patients, the numbers of Tfr cells are significantly increased in both the blood and submandibular glands when compared with those levels in peripheral blood and tonsils from healthy individuals [40, 79]. The percentage of these cells is positively correlated with other clinical parameters, including serum IgG4 levels and the number of involved organs in IgG4-RD patients, indicating the possible involvement of Tfr cells in IgG4-specific class-switch recombination in lesions of IgG4-RD [40, 79]. Furthermore, the number of IL-10-producing circulatory Tfr cells in IgG4-RD patients is increased compared with that in healthy elderly individuals, promoting B cell differentiation and GC formation [40, 79].

#### 2.9.6. Circulating PD-1^hi^CXCR5^−^Peripheral T Cells (Tph) in IgG4-RD

*(1) Characteristics and Functions of Tph in IgG4-RD*. A recent study on some autoimmune diseases exposed an unidentified subset of CD4^+^ T cells, named Tph cells (PD-1^hi^CXCR5^−^CD4^+^ Tph cells) [80]. These cells exhibit Tfh cell-like features, including secretion of IL-2. In addition, Tph cells have a special expression profile of chemokine receptors, such as CCR2, CCR5, CXCL13, and CX3CR1, which promote their migration to inflamed sites [80]. CXCL13, expressed by these cells, may enlist CXCR5-expressing immune cells, including B cells and Tfh cells, to initiate and maintain fibroinflammation, that is, with the aid of Tph, Tfh cells and B cells are recruited to the site of inflammation to assist in the formation of ectopic lymphoid structures at these sites [6, 40, 67, 81–84]. Furthermore, Tph cells express high granzyme A granules levels, which are cytolytic granules that activate programmed cell death, and when these cells are activated they will exert their cytotoxic activity through the release of these granules into the target cells [20, 83].

*(2) Tph Cells in IgG4-RD*. In IgG4-RD patients, there are increased levels of the circulating PD-1^high^ CXCR5^−^CD4^+^ Tph and the percentage of these PD-1^hi^CXCR5^−^CD4^+^ Tph cells is positively correlated with serum levels of IgG4 and soluble IL-2 receptors and with the number of involved organs in IgG4-RD patients [20, 40, 83]. According to their capacity to initiate and maintain fibroinflammation, PD-1^hi^CXCR5^−^CD4^+^ Tph cells may play a more pathogenic role than their Tfh counterparts in IgG4-RD pathogenesis [82]. Treatment with glucocorticoids leads to a decline in Tph cells, suggesting that Tph could be involved in IgG4-RD pathogenesis [83].

#### 2.9.7. CD4^+^SLAMF7^+^ Cytotoxic T Cells (CD4+CTLs) in IgG4-RD

*(1) Immunobiology of CD4+ SLAMF7+CTLs*. Cytotoxic CD4^+^ T cells (CD4+CTLs) are classically known as the CD28^low^ subpopulation among CD4+T cells [85]. These cells are characterized by their abilities to secrete granzymes A and B and perforin in order to kill target cells through the recognition of MHC class II-restricted antigen presentation [40, 43, 86].

Moreover, these cells possess a surface protein called SLAMF7 and can secrete Th1-like proinflammatory and profibrotic cytokines, including IL-1*β*, TGF-*β*1, and IFN-*γ*, suggesting their role in tissue inflammation and fibrosis in various immune responses [40, 86, 87].

*(2) CD4^*+*^SLAMF7^*+*^ CTLs in IgG4-RD*. In IgG4-RD, CD4^+^ CTLs infiltrate the affected tissue and increased in peripheral circulation and at these sites CD4^+^ CTLs show oligoclonal expansion [43, 55]. The profibrotic cytokines TGF-*β*, IL-1*β*, and IFN-*γ* secreted by CD4^+^ CTLs may play a role in tissue fibrosis in IgG4-RD [55].

Both CD4^+^ CTLs and CD8^+^ CTLs, but not Th1 and Th2 cells, are significantly more abundant in the lesions of IgG4-RD, correlate with disease activity, and may contribute to the initiation of T cell-mediated apoptosis and tissue fibrosis in the disease-affected organs [29, 88]. Saeki et al. revealed that the level of CTLs correlates with the number of IgG4-RD affected organ [89].

In IgG4-RD patients, the clonally expanded populations of CD4^+^ CTLs and their suppression following glucocorticoid administration suggest that these cells may play a central role in disease development [55, 90].

#### 2.9.8. CD8^+^PD-1^+^ Cytotoxic T Cells in IgG4-RD

*(1) Immunobiology of CD8^+^PD-1^+^ CTLs*. Cytotoxic CD8+ T cells exert their cytotoxic activity by releasing two types of cytotoxic proteins. The first protein type is granzymes (A and B), which are able to induce apoptosis in any type of target cell, whereas the second protein type is the pore-forming protein perforin, which pierces holes in the target cell membrane, thus allowing the granzymes to enter the cell and exert their cytotoxic activity through apoptosis [91].

*(2) CD8^*+*^PD-1^*+*^ CTLs in IgG4-RD*. Little information exists in the literature regarding the role of CD8^+^ CTLs in the pathogenesis of IgG4-RD.

Ohta et al. (2012) revealed a significant increase in the number of IFN-*γ*^+^CD8^+^ T (Tc1) cells in peripheral blood, although there were no differences in the percentage of IL‐4-expressing CD8^+^ T (Tc2) subsets between patients with IgG4‐related sclerosing disease and healthy controls [92].

Perugino et al. found that CD8^+^ CTLs are correlated with disease activity and may initiate T cell-mediated apoptosis and tissue fibrosis in the disease-affected organs [88].

## 3. IgG4-RS: Küttner's Tumor and Mikulicz's Disease

Generally, IgG4-RD are lymphoproliferative disorders associated with hyper-IgG4 (gammaglobulinemia) and IgG4-producing plasma cell expansion with fibrotic or sclerotic changes in the disease-affected organs. IgG4-RD include a wide variety of diseases, such as Mikulicz's disease, Küttner's tumor, autoimmune pancreatitis (AIP), interstitial nephritis, retroperitoneal fibrosis, and prostatitis [93].

Mikulicz's disease and Küttner's tumor have been reported to be associated with elevated serum IgG4 levels and prominent infiltration of plasmacytes expressing IgG4 with occasional storiform fibrosis [21, 93, 94].

Küttner's tumor and Mikulicz's syndrome are two similar disorders that share common features and are now considered a type of IgG4-RD [3, 95–97]. The histology of Küttner's tumor indicates severe fibrosclerotic lesions with infiltration of IgG4^+^ plasma cells, whereas the fibrosis is less severe in Mikulicz's syndrome. Therefore, it is difficult to differentiate between the two diseases histologically [3, 96, 97].

Recently, authors have suggested that Küttner's tumor and Mikulicz's disease may in fact represent two variants of IgG4-associated sialadenitis [12, 38].

## 4. Küttner's Tumor (IgG4-Related Sialadenitis) and T Cells

### 4.1. Küttner's Tumor (IgG4-RS)

Küttner's tumor, sometimes identified as chronic sclerosing sialadenitis, is a fibroinflammatory process of underrecognized cause that involves the submandibular gland of patients with IgG4-RD [95, 98, 99].

It is a relatively uncommon lesion that is considered an IgG4-RD because of its histologic features and elevated serum IgG4 levels [4, 95]. Moreover, it is characterized by dense lymphocytic infiltration, dilated ducts, progressive periductal fibrosis, lymphoid follicle formation, acinar atrophy, and eventually marked sclerosis of the affected gland [100, 101].

According to the literature, unilateral submandibular gland involvement, previously known as Küttner's tumor, is now preferably termed as IgG4-RS of the submandibular gland [18, 93]. In contrary to that, Marcus et al. revealed that, in Küttner's tumor, the mass-forming lesions of the salivary glands with histopathologic features characteristic of chronic sclerosing sialadenitis may not always be indicative of an immune-mediated reaction and are not always associated with IgG4 overexpression. This suggests that this condition may have many etiologies, one of which is IgG4-RD [102].

### 4.2. Etiology and Theories of Origin of Küttner's Tumor

The exact etiology of this entity is unclear. There are several theories that have been presented to explain its origin [93, 98, 103, 104].

The first theory identified sialolithiasis as an etiological factor. Sialolithiasis induces inflammation and fibrosis and has been demonstrated in 29%–83% of cases of chronic sclerosing sialadenitis [98]. Some authors have suggested that stones lead to the obstruction of salivary flow [98]. Secretion stasis induces inflammation and fibrosis, resulting in chronic sclerosing sialadenitis. However, others have suggested that the formation of calculi is secondary to sialadenitis [98].

The second theory indicates that this disease is caused by infectious agents, such as ascending microbial infection of the oral cavity, or that it is a result of foreign bodies, such as duct obstruction by foreign bodies [98, 104].

The third theory is that it is an active local immune reaction in which there is involvement of IgG4 Abs [93, 98].

The fourth theory indicates a group of etiological factors and mechanisms which assume secretory dysfunction with ductal inspissation, duct abnormalities, or autoimmune reaction as a cause of Küttner's tumor [104].

### 4.3. Findings That Support Autoimmunity in Küttner's Tumor

Küttner's tumor shows features of immune compartment dysregulation, where the number of CD4^+^ T helper cells significantly exceeds that of CD8^+^ cytotoxic T cells and the quantity of CD4^+^ and CD8^+^ cells is higher than that of CD3^+^ cells. Additionally, there is the formation of tertiary lymphoid follicles in which CD3^+^ T cells are located at of the follicle periphery [103, 105]. Furthermore, B lymphocytes (CD19^+^ and CD20^+^) could be found in tertiary lymphoid follicles [105].

In the salivary glands of Küttner's tumor, there are abundant cytotoxic T cells that are found in the parafollicular zone of germinal centers and in interfollicular areas and centered mostly around ducts and between acini that are intimately related to epithelial cells. These cytotoxic T cells show monoclonal and oligoclonal expansion [103, 105]. In addition to that, the number of the cytotoxic T cells decreased with the increase in the sclerosis [98].

### 4.4. Findings Supporting Küttner's Tumor as IgG4-Related Sialadenitis

Salivary gland specimens collected from Küttner's tumor patients show IgG4-producing cells (IgG4^+^ plasmacytes), and the peripheral blood shows an increased level of serum IgG4 [106]. Immunohistochemical studies showed that the percentage of IgG/IgG4-producing cells is more than 45% of the total IgG-producing cells in Küttner's tumor [93].

Many cases of Küttner's tumor are associated with sclerosing lesions in the extra-salivary gland tissues as multiple organs are affected [93]. The extra-salivary gland lesions in Küttner's tumor patients are complicated by autoimmune pancreatitis (AIP) and IgG4 immune related tubulointerstitial nephritis [94].

Steroid treatment leads to rapid improvement in glandular swelling, reduction in the number of IgG4-producing plasma cells in the salivary gland lesions, and restoration of the salivary gland function [99, 107].

### 4.5. Clinical Features of Küttner's Tumor

Küttner's tumor is clinically characterized by a firm, sometimes painful, mass in the submandibular glands. It may occur unilaterally or bilaterally, predominantly involving the submandibular gland, but it can also occur in other major and minor salivary glands, including the parotid gland. Additionally, it has been reported to involve both the submandibular and parotid glands simultaneously [98, 104, 108, 109].

As it often could not be distinguished from a true salivary gland neoplasm because of its clinical similarity, this disease had been referred to as a tumor [110], but with the advancements of diagnosing methods it has been reclassified as a tumor-like lesion [104, 111].

### 4.6. Histology of Küttner's Tumor

The histopathological features of Küttner's tumor may progress through four stages based on the progressiveness and severity of inflammation [112].

Stage 1 includes a mild, focal chronic lymphoplasmacytic cell infiltration, primarily in the periductal area with little periductal fibrosis. The glandular components are preserved. Stage 2 exhibits denser lymphocytic infiltration and increased periductal fibrosis is present. Stage 3 shows more prominent lymphoplasmacytic infiltrate with reactive tertiary lymphoid follicle formation, extensive fibrosis with acinar atrophy, periductal hyaline deposition, and ductal dilatation. Stage 4 is evident by destruction of lobular components accompanied by extreme sclerosis and severe loss in the parenchyma.

### 4.7. Immunohistochemical Studies of Küttner's Tumor

Immunohistochemical studies of Küttner's tumor specimens of the salivary glands have revealed the abundance of B cells and T cells in the examined tissue specimens [98, 103, 108, 113–115].

### 4.8. The Diagnostic Criteria of IgG4^+^ Küttners Tumor

Unlike in Mikulicz's disease, there are no unified or specified criteria approved for the diagnosis of Küttner's tumor as an IgG4-RS, but, for the diagnosis of this disease as an IgG4-RS, all authors depended on three comprehensive diagnostic criteria items for IgG4-RD, in particular the presence and the ratio of IgG4 in the affected salivary glands and peripheral circulation.

### 4.9. T Cells and T Cells Subsets in the Pathogenesis of Küttner's Tumor as IgG4-RS

In general, the affected gland tissues show an abundance of CD3^+^, CD4^+^, and CD8^+^ T cells [98, 103, 116–119].

### 4.10. T Cell Subsets: Features and Findings in Küttner's Tumor (IgG4-RS)

#### 4.10.1. T Helper and Cytotoxic T Cells in IgG4-RS

In IgG4-RS, CD3^+^, CD4^+^, and CD8^+^ T cells are the predominant cells that infiltrate the affected gland [103, 114, 116–118]. The ratio of CD4^+^ to CD8^+^ T cells is 1 : 2, and the percentage of CD4^+^ and CD8^+^ cells is slightly greater than that of CD3^+^ cells [103].

#### 4.10.2. CD3^+^ T Cells in IgG4-RS

The glandular inflammatory infiltrate shows abundant CD3^+^ lymphocytes in the affected gland, particularly in the intraepithelial sites [120–123]. Also, these cells might be found at the periphery of tertiary lymphoid follicles or perifollicular zone [98, 103, 104, 116, 121, 122].

Consuegra et al. (2007) found that CD3^+^ T cells infiltrate primarily in the periductal area [124].

#### 4.10.3. CD4^+^ T Helper Cells in IgG4-RS

In the affected gland, CD4^+^ T helper cells are predominantly infiltrate around the glandular ducts and acini [108, 114]. Some CD4^+^ T helper cells localize to the lymphoid follicles in the affected gland, and the percentage of CD4^+^ T helper lymphocytes is larger than that of CD8^+^ CTLs [103].

#### 4.10.4. CD4^+^GATA3^+^ Th2 and CD4^+^FoxP3^+^ Treg Cells in IgG4-RS

Both CD4^+^GATA3^+^ Th2 and CD4^+^FoxP3^+^ Treg cells are predominant and highly active in the affected gland [3, 4, 12, 44–46, 125].

In IgG4-RS, it is postulated that the inflammatory and fibrotic processes are derived by Th2 and Treg cells secreted cytokines. This is contrary to most autoimmune disorders, where polarized Th1 and/or Th17 subsets are responsible for the inflammatory process [4, 12, 16, 125].

#### 4.10.5. CD4^+^SLAMF7^+^ Cytotoxic T Cell in IgG4-RS

CD4^+^SLAMF7^+^ CTL cells are found in the parafollicular zone of germinal centers and in interfollicular areas of the affected gland, especially near ducts and between acini that are intimately close to the epithelial cells [101, 103, 105, 108]. The infiltrated populations of CD4^+^ CTLs show monoclonal and oligoclonal expansion [103, 108, 126].

The presence of cytotoxic T cells intimately close to the ductal epithelial cells indicates that IgG4-RS may be the consequence of an immune process triggered by intraductal epithelial agents [101, 103, 105, 108, 127].

In stages 2 and 3 of disease progression, there is intensive infiltration by periductal and intraepithelial CD4^+^ CTLs [103], whereas, in stages 3 and 4, there is an increase in fibrosis which causes a decline in the number of cytotoxic T cells [103]. Finally, the interactions between B cell and the CD4^+^SLAMF7^+^ CTLs result in tissue inflammation and fibrosis [17].

#### 4.10.6. CD8^+^PD-1^+^ Cytotoxic T Cells in IgG4-RS

There is an abundant infiltration of granzyme A^+^ and granzyme B^+^ CD8^+^ T cells in the affected submandibular gland [103, 114, 128].

## 5. Mikulicz's Disease (IgG4-RDS)

In defining and differentiating Mikulicz's disease as an IgG4-RDS, Stone et al. suggested the disposal of the term “Mikulicz's disease” when referring to IgG4-RD patients with involvement of the lacrimal, parotid, and submandibular glands and to instead use terms that refer to the specific individual organ involved, such as IgG4-related parotitis for those with parotid gland disease, IgG4-RS or IgG4-related submandibular gland disease, which is also known as Küttner's tumor, for those with submandibular gland involvement, IgG4-RDS, which is also known as Mikulicz's disease, for those with bilateral and symmetrical enlargement of both lacrimal and salivary glands, and IgG4-related dacryoadenitis for those with lacrimal gland disease only [18].

### 5.1. The Etiology and Pathogenesis of Mikulicz's Disease

The etiology of Mikulicz's disease, similar to that of other IgG4-RDs, is still unclear, and little is known regarding its etiology and pathogenesis. Some authors consider its etiology and pathogenesis to be controversial [67, 129, 130].

In the pathogenesis of Mikulicz's disease, most of the infiltration of IgG4^+^ cells in the affected glands and peripheral blood is polyclonal, revealing that Mikulicz's disease is an IgG4-RD involving multiple organs [129].

### 5.2. The Diagnostic Criteria of IgG4^+^ Mikulicz's Disease

In Mikulicz's disease, some patients did not show typical symptoms involving the lachrymal, parotid, or submandibular glands, but they exhibited elevated serum IgG4 levels; therefore, a unified set of criteria have been approved for the diagnosis of Mikulicz's disease, which is shown in Table 3 [131].

### 5.3. Clinical Pictures of Mikulicz's Disease

Mikulicz's disease was once considered a subtype of Sjögren's syndrome because of the histopathological similarities between the two diseases. However, recent studies demonstrated that Mikulicz's disease is a distinct entity and should be regarded as an IgG4-RD [14, 21, 94, 132, 133].

Clinically, Mikulicz's disease is characterized by symmetrical, bilateral, painless swelling of the lacrimal, parotid, and submandibular glands. Furthermore, it is characterized by high serum IgG4 concentrations and abundant infiltration of IgG4^+^ B cells in the lacrimal and salivary glands [3, 14, 21].

### 5.4. Complications Associated with Mikulicz's Disease

Complications of Mikulicz's disease include AIP, retroperitoneal fibrosis, and tubular interstitial nephritis [14]. Some patients with Mikulicz's disease have complained of olfactory disturbance [134].

Himi et al. found an abundance of IgG4^+^ plasmacytes in the nasal mucosa of Mikulicz's disease patients who complained of olfactory disturbance, suggesting that these disturbances could be related to this mucosal infiltration of IgG4^+^ B cells [106].

### 5.5. T Cells in the Immunopathogenesis of Mikulicz's Disease (IgG4-RDS)

Similar to other IgG4-RDs, Mikulicz's disease is characterized by elevated serum IgG4 levels, tissue infiltration by IgG4^+^ plasma cells, and storiform fibrosis [3, 14, 18, 19]. Unlike Sjögren's syndrome, Mikulicz's disease is characterized by nonperiductal lymphocytic infiltration with hyperplastic multiple germinal center formation and mild destruction of the acini. Some authors considered Küttner's tumor a subtype of Mikulicz's disease confined primarily to the submandibular salivary glands with predominance of fibrosis (sclerosing sialadenitis) and salivary duct destruction [135].

The underlying pathogenesis of IgG4-RDS remains largely unidentified [67, 129, 130].

In IgG4-RDs, large numbers of T cells are present at the affected organs, including the salivary glands, indicating that T cells are involved in the pathogenesis of this disease. Evidence of the T cells involvement in all IgG4-RDs, including IgG4-RDS, has been increasing, and recently the roles of T cell subsets in IgG4-RD pathogenesis have been largely clarified [6].

The cytokine profile of Mikulicz's disease shows that Th2 and Treg cells are predominant [45, 136]. In addition, IL-21, which is secreted by cytotoxic T cells, is overexpressed in Mikulicz's disease and is involved in germinal center formation and IgG4 class-switching [14, 137–140].

Most previous studies on the contribution of T cells to the pathogenesis of IgG4-RDs have focused on Th2 and Treg cells as the dominant cells that infiltrate the affected tissue; thus, these two subtypes are considered as the major T cells that play a role in the pathogenesis of IgG4-RDS.

Additionally, recent studies identified new subsets of T cells, such as T follicular cells and CD4^+^ CTLs, in the affected tissue that may also play a role in pathogenesis [55, 141].

### 5.6. T Cells Subsets: Features and Findings in Mikulicz's Disease (IgG4-RDS)

#### 5.6.1. CD4^+^ and CD8^+^ T Lymphocytes in General

In Mikulicz's disease, the affected gland tissue shows infiltration of CD4^+^ T helper cells and CD8^+^ CTLs [142].

Immunobiologically, cytolytic T cells destroy tissue through perforin or Fas lytic pathways [142–144]. In Mikulicz's disease, the infiltrated lymphocytes do not express large amounts of Fas and Fas-L. This limited expression of Fas and Fas-L in the glandular tissue of Mikulicz's disease patients may clarify why the intense lymphocyte infiltration does not cause major destruction of acinar cells [142].

#### 5.6.2. Classical Th1 in IgG4-RDS

The levels of Th1 cytokines IFN-*γ* and IL-12 are utilized as indicators to study the activity of classical Th1 [145]. In the submandibular salivary gland of Mikulicz's disease (IgG4-RDS) patients, the levels of these cytokines show no significant changes when compared with their levels in healthy individuals [145].

#### 5.6.3. Nonclassical Th17/Th1 (Th1 Producing IL-17, Also Called Nonclassical Th1) in IgG4-RDS

Th17 cells, which primarily produce IL-17, have been suggested to play a critical role in the pathogenesis of autoimmune diseases. Th17 is a very flexible phenotype of T cells because it can easily shift to Th1 phenotype in inflammatory sites via the activity of proinflammatory cytokines, such as IL-12. This newly produced Th1 is designated as a Th17-derived Th1 cell or nonclassical Th1 that has the capacity to produce IL-17. Nonclassical Th1 cells play a more important role than classical Th1 and even Th17 cells in the persistence of chronic inflammation in several autoimmune disorders [133]. However, the role of IL-17 in Mikulicz's disease currently remains unknown.

Yamamoto et al. reported that the IFN-*γ*/IL-4 ratio is very high in Mikulicz's disease, and the elevated IgG4 production is considered dependent upon the high level of IFN-*γ*/IL-4 [132].

In the submandibular salivary gland and the lymph node of Mikulicz's disease patients, Nanke et al. detected IFN-*γ*^+^, IFN-*γ*^+^IL-17^+^, and IL-17^+^ cells, which are proposed to be Th17/Th1 cells, suggesting that Th17/Th1 cells may be involved in the pathogenesis of Mikulicz's disease [133].

#### 5.6.4. CD4^+^GATA3^+^ Th2 Cells in IgG4-RDS

*(1) CD4^*+*^GATA3^*+*^ Th2 Cells in the Salivary Gland of IgG4-RDS*. On comparing Mikulicz's disease patients with healthy individuals, CD4^+^GATA3^+^ Th2 cells are predominant and significantly increased in the affected salivary glands of Mikulicz's disease patients [45–47, 55, 136, 145]. This increase is accompanied by significant increase in the mRNA expression levels of Th2 cytokines including IL-4, IL-10, IL-13, and IL-21 which may serve key roles in the development of the disease and are responsible for the occurrence of symptoms and signs [45–47, 145]. Zen et al. revealed that the expression of Th2 cytokines IL-4 and IL-10 is positively correlated with the ratio of IgG4^+^/IgG^+^ cells in tissues with IgG4-RDS indicating their close association with IgG4 production [45]. Also, In IgG4-RDS, the overexpression of IL-21 by Th2 cells participates in inducing the formation of multiple germinal centers and IgG4 production [137].

Furthermore, predominant infiltration of Th2 cells and increased levels of their cytokines in the salivary glands of patients with Mikulicz's disease could suppress production of IFN-*γ* from Th1 cells, which may prevent the destruction of the glandular acinar structure [146].

*(2) CD4^*+*^GATA3^*+*^ Th2 Cells in Peripheral Circulation of IgG4-RDS*. CD4^+^ Th2 cell represents the predominate phenotype of CD4^+^ in the peripheral circulation of IgG4-RDS [49, 145].

*(3) Association of Th2 and Atopy in IgG4-RDS*. Allergic immune responses are induced by allergen-specific Th2 cytokines, such as IL-4 and IL-13, which promote the secretion of IgG4 and IgE by B cells. In particular, IL-4 directs naïve B cells to switch to producing IgG4 and IgE [147, 148]. IgG4-RDS patients frequently have a history of bronchial asthma and allergic rhinitis with severe eosinophilia and elevated serum IgE levels [45, 149]. In IgG4-RDS, CD4^+^GATA3^+^ Th2 cells were shown to be relatively rare in patients without associated allergic diseases [145]. Mattoo et al. reported that circulating memory Th2 cells in IgG4-RD were detected in a limited population of subjects with atopy [41].

#### 5.6.5. Th1/Th2 Balance in IgG4-RDS

Several studies have revealed that autoimmune diseases are caused by disruption of Th1/Th2 balance [49, 150].

Most studies revealed that Th1/Th2 balance toward Th2 resulted in the overexpression of Th2 cytokines IL-4 and IL-10 along with IgG4 production in the labial salivary glands of patients with IgG4-RDS [45, 151].

Contrary to previous studies, Ohta et al. (2012) reported a strong predominance of Th1 and cytotoxic type cells in the salivary glands of IgG4-RDS patients, suggesting that this disruption of the Th1/Th2 balance might be because of differences in the specimens examined or the severity of the disease [92].

Generally, Th1/Th2 balance in peripheral CD4^+^ T cells of IgG4-RDS patients deviates toward Th2 and the expression of Th2-type cytokines is elevated, indicating that IgG4-RDS has a Th2-predominant phenotype [50, 145]. Another reason to consider Mikulicz's disease a Th2-predominant immune reaction is the high serum IgG4 and IgE levels which accompany the occurrence of the increased level of Th2 cytokines in Mikulicz's disease patients [49, 50, 145].

#### 5.6.6. Th17 Cells in IgG4-RDS

Th17-related cytokines and molecules are rarely expressed in patients with Mikulicz's disease [137, 152].

#### 5.6.7. CD4^+^CD25^+^Foxp3^+^ Treg Cells in IgG4-RDS

*(1) Tissue Treg Cells in IgG4-RDS*. In IgG4-RDS, the activation of Treg cells in IgG4-RD is reported to be based on the increased expression of FoxP3 messenger RNAs in the affected tissues when compared with its decreased expression in classic autoimmune diseases [42, 45, 47].

In the affected gland in IgG4-RDS patients, most of the studies revealed that there are an increased number of CD4^+^Foxp3^+^ Treg cells which are predominantly located around the acinar and ductal cells [45, 47, 130, 136, 152, 153].

*(2) Circulatory Treg Cells in IgG4-RDS*. Increased numbers of CD4^+^CD25^high^ Treg cells are observed in the circulation of IgG4-RDS patients [42, 64].

*(3) Treg Cell Molecules and Cytokine Levels and in IgG4-RDS*. The expression levels of IL-4, IL-5, IL-10, TGF-*β*, and FoxP3 mRNA in labial salivary glands of patients with Mikulicz's disease have been shown to be higher than the levels in healthy persons [45]. IL-4, IL-10, and FoxP3 are positively and strongly correlated with the ratio of IgG4 : IgG, suggesting that these cytokines produced by Treg cells are involved in the class switching of B cells to IgG4-roducing cells [45, 154]. In the glands of IgG4-RDS patients, the Treg cytokine TGF-*β* level is upregulated and appears to play central fibrogenic roles in tissue fibrosis [18, 45, 154, 155].

Corticosteroid treatment improving the disease activity of IgG4-RDS is accompanied by a decline in the frequency of circulating Treg cells [154]. Akiyama et al. [154] reported two possibilities to be considered for the decrease in the frequency of circulating Treg cells after systemic corticosteroid treatment. The first possibility is that Treg cells might be secondarily reduced as a result of the direct effect of corticosteroids. The second suggests that Treg cells might decline with the reduction in the disease activity of IgG4-RDS.

#### 5.6.8. CD4^+^CXCR5^+^ Tfh Cells in IgG4-RDS

Tfh cells are identified as a CD4^+^ Th phenotype, expressing high levels of the chemokine receptor CXCR5. These cells are reported to control the activity of CD4^+^ effector Th cells, B cell activation, and differentiation and promote ectopic germinal centers formation by IL-21, which is secreted by CD4^+^ Th cells, including Th2, Th17, and especially Tfh cells [156–158].

*(1) Tissue CD4^*+*^CXCR5^*+*^ Tfh Cells in IgG4-RDS*. In IgG4-RDS, Maehara et al. revealed that an increase in the expression of some Tfh cell-related genes in IgG4-RDS-affected tissue resulted from increased differentiation and activation of Tfh cells [43].

IgG4-RDS shows a high frequency of germinal center formation in the affected salivary glands as a result of increased differentiation and activation of Tfh cells [137].

*(2) Peripheral Circulation Tfh Cells in IgG4-RDS*. In IgG4-RDS, there is positive correlation between circulating type of Tfh (cTfh2) and the number of plasmablasts because cTfh2 cells have the efficiency to induce differentiation of naïve B cells into plasmablasts [66].

*(3) Tfh Cells and Their Cytokine Levels in IgG4-RDS*. The increased level of Tfh molecules and cytokines supports the role of Tfh cells in the progression of disease as a lymphoproliferative disorder, particularly in the formation, growth, and activation of ectopic germinal centers in IgG4-RDS patients [152].

Tfh-related molecules, CXCR5, B cell lymphoma 6 protein (Bcl-6), and IL-21 are highly expressed on infiltrating lymphocytes in or around the ectopic germinal centers of labial salivary gland lesions from IgG4-RDS patients [137, 151].

In IgG4-RDS, the serum level of IL-4 Tfh-related cytokine is increased and positively correlated with the serum IgG4 level or the IgG4 : IgG ratio [66].

IL-21, which is one of the cytokines produced by Tfh, may be involved in the class-switching of IgG4, thus affecting the IgG4/IgG ratio in IgG4-RDS [159–161]. The expression of this cytokine in the labial salivary glands is shown to be positively correlated with the number of germinal centers formed the in patients with IgG4-RDS [137].

#### 5.6.9. T Follicular Regulatory (Tfr) Cells in IgG4-RDS

In IgG4-RDS patients that show the involvement of salivary glands, the numbers of Tfr cells in circulating peripheral blood and infiltrating the submandibular glands are significantly increased compared with those in healthy individuals [79].

#### 5.6.10. CD4^+^ Granzyme A (GZMA)^+^SLAMF7^+^ CTLs in IgG4-RDS

*(1) Tissue Infiltration of CD4^*+*^GZMA^*+*^SLAMF7^*+*^ CTLs in IgG4-RDS*. In IgG4-RDS tissues, nine genes associated with CD4^+^ CTLs are overexpressed; in particular, the mRNA expression of GZMA was shown to be significantly higher in submandibular specimens from patients with IgG4-RD compared with that in corresponding tissues from healthy individuals [43].

Some studies revealed that CD4^+^GZMA^+^ CTLs, but not Th1 and Th2 cells, are significantly more abundant in the lesions of patients with IgG4-RDS than in healthy individuals, suggesting that the activity of IgG4-RDS is prominently linked to tissue infiltration by CD4^+^GZMA^+^ CTLs but not to Th1 and Th2 cells. Also, the increased IFN-*γ* expression in IgG4-RDS tissues is more likely from CD4^+^ CTLs not from Th1 cells [43, 55]. Furthermore, the ratio of CD4^+^GZMA^+^ CTLs in submandibular glands from patients with IgG4-RDS is positively correlated with serum IgG4 concentrations and the number of affected organs.

Regarding the distribution of these cells within the specimen of affected organ, it has been found that CD4^+^TGF-*β*1^+^ cells are abundantly present in areas where fibrotic pathology is seen in IgG4-RDS [43, 55]. This is supporting the suggestion that IL-1*β* and TGF-*β*, secreted by CD4^+^SLAMF7^+^ CTLs, may contribute to fibrogenesis in IgG4-RDS [43, 55].

*(2) Circulating CD4* ^*+*^ *GZMA* ^*+*^ *CTLs in IgG4-RDS*. Maehara et al. described clonally expanded circulating CD4^+^GZMA^+^ CTLs in IgG4-RDS with predominant infiltration in the affected tissues where they secrete IFN-*γ*, IL-1*β*, and TGF-*β*1 [43, 55].

CD4^+^ CTLs have been shown to decrease in response to B cell depletion therapy, which may reflect the interaction between CD4^+^ CTLs and B cells in the pathogenesis of IgG4-RDS. This interaction may be explained by the potential of some activated B cells to present antigens to tissue-infiltrating CD4^+^ CTLs, causing their activation and subsequently exerting their cytotoxic activity [55, 141].

#### 5.6.11. CD8^+^ CTLs in IgG4-RDS

In relation of CD8^+^ CTLs, Tsubota et al. revealed increased numbers of these cells in the affected organs [142]. Tabeya et al. showed that PD-1^+^CD8^+^ CTLs are mostly located around the lymphoid follicles in IgG4-RDS [130].

## 6. Conclusions

The immunopathogenesis of IgG4-RS (Küttner's tumor and Mikulicz's disease) as a type of IgG4-RD has not yet been fully clarified. Immunohistological studies have revealed that IgG4-RS lesions are characterized by the abundant infiltration of different T cell subsets in the salivary tissues and peripheral blood.

Recent studies have abundantly clarified that T cells and their interactions with and activation of B cells play key roles in the pathogenesis of IgG4-RS.

Further research works are required to elucidate the roles of the T cell subsets and their secreted cytokines in the complex mechanisms of IgG4‐RS pathogenesis, which could lead to the development of novel pharmacological strategies aimed to control the overactivity of T and B cells and their cytokine network, thereby inhibiting the initiation and/or progression of IgG4‐RS.

## Figures and Tables

**Table 1:** The 2020 revised comprehensive diagnostic (RCD) criteria for IgG4-related disease (IgG4-RD) proposed by Umehara et al. [31].

1. Clinical and radiological features	One or more organs show diffuse or localized swelling or a mass or nodule characteristic of IgG4-RD. In single organ involvement, lymph node swelling is omitted.
2. Serological diagnosis	Serum IgG4 levels greater than 135 mg/dl.
3. Pathological diagnosis	Positivity for two of the three following criteria:
	1. Dense lymphocyte and plasma cell infiltration with fibrosis.
	2. Ratio of IgG4^+^ plasma cells/IgG^+^ cells greater than 40% and the number of IgG4^+^ plasma cells greater than 10 per high powered field
	3. Typical tissue fibrosis, particularly storiform fibrosis, or obliterative phlebitis
Diagnosis	Definite: 1, 2, and 3
	Probable: 1 and 3
	Possible: 1 and 2

**Table 2:** T cell subsets involved in the pathogenesis of IgG4-related disease.

Class	T cell subsets
T cells	CD4^+^GATA3^+^ T helper 2 cells
	CD4^+^FoxP3^+^ T regulatory cells
	CD4^+^CXCR5^+^ T follicular helper cells
	CD4^+^ SLAMF7^+^cytotoxic T lymphocytes
	—PD-1^hi^CXCR5^−^ peripheral T helper-like cells
	—T follicular regulatory cells

**Table 3:** Diagnostic criteria of IgG4^+^ mikulicz's disease (approved by the Japanese Society for Sjögren's Syndrome 2008) [131].

Symmetrical swelling of at least two pairs of lachrymal, parotid, or submandibular glands for at least 3 months
AND Elevated serum IgG4 (>135 mg/dL)
OR Histopathological features including lymphocyte and IgG4+plasma cell infiltration (IgG4^+^ plasma cells/IgG^+^ plasma cells > 50%) with typical tissue fibrosis or sclerosis.

## Data Availability

No data were used to support the findings of this study.

## Abbreviations

IgG4-RD: Immunoglobulin G4-related disease
IgG4-RS: IgG4-related sialadenitis
IgG4-RDS: IgG4-related dacryoadenitis and sialadenitis
IgG4+: IgG4 positive
Tfh: Follicular helper T
Treg cells: Regulatory T cells
Tph: Peripheral T helper
TGF-*β*: Transforming growth factor-*β*
IL-: Interleukin-
IFN: Interferon
GC: Germinal center
Abs: Antibodies.
